# Pyridylnidulin exerts anti-diabetic properties and improves non-alcoholic fatty liver disease in diet-induced obesity mice

**DOI:** 10.3389/fmolb.2023.1208215

**Published:** 2023-06-22

**Authors:** Sutharinee Likitnukul, Surapun Tepaarmorndech, Theerayuth Kaewamatawong, Arunrat Yangchum, Chanathip Duangtha, Pimrapat Jongjang, Supachoke Mangmool, Darawan Pinthong, Masahiko Isaka

**Affiliations:** ^1^ Department of Pharmacology, Faculty of Science, Mahidol University, Bangkok, Thailand; ^2^ Faculty of Medicine, Chulalongkorn University, Bangkok, Thailand; ^3^ Department of Pathology, Faculty of Veterinary Science, Chulalongkorn University, Bangkok, Thailand; ^4^ National Center for Genetic Engineering and Biotechnology (BIOTEC), National Science and Technology Development Agency (NSTDA), Pathumthani, Thailand

**Keywords:** anti-diabetic, diabetes, non-alcoholic fatty liver disease, pyridylnidulin, obesity, metabolic disorders

## Abstract

**Introduction:** Non-alcoholic fatty liver disease (NAFLD) is one of the metabolic disorders related to the pathophysiology of type 2 diabetes mellitus (T2DM). Therapeutic strategies are focused on the improvement of energy balance and lifestyle modification. Additionally, the derivative of the bioactive fungal metabolite is of interest to provide health benefits, especially in obese and pre-diabetic conditions. In our screening of anti-diabetic compounds from fungal metabolites and semisynthetic derivatives, a depsidone derivative, namely pyridylnidulin (PN), showed potent glucose uptake-inducing activity. The present study aimed to investigate the liver lipid metabolism and anti-diabetic properties of PN in diet-induced obesity mice.

**Methods:** Male C57BL/6 mice were induced obesity and pre-diabetic conditions by dietary intervention with a high-fat diet (HFD) for 6 weeks. These obese mice were orally administered with PN (40 or 120 mg/kg), metformin (150 mg/kg), or vehicle for 4 weeks. Glucose tolerance, plasma adipocytokines, hepatic gene and protein expressions were assessed after treatment.

**Results:** Improved glucose tolerance and reduced fasting blood glucose levels were found in the PN and metformin-treated mice. Additionally, hepatic triglyceride levels were consistent with the histopathological steatosis score regarding hepatocellular hypertrophy in the PN and metformin groups. The levels of plasma adipocytokines such as tumor necrosis factor-α (TNF-α) and monocyte chemoattractant protein-1 (MCP-1) were reduced in the PN (120 mg/kg) and metformin-treated mice. In addition, hepatic gene expression involved in lipid metabolism, including lipogenic enzymes was significantly reduced in the PN (120 mg/kg) and metformin-treated mice. The increased protein expression levels of phosphorylated AMP-activated protein kinase (p-AMPK) was also found in PN and metformin-treated mice.

**Discussion:** Considering the increased p-AMPK protein expression levels in PN and metformin-treated mice were revealed as the underlying mechanisms to improve metabolic parameters. These results suggested that PN provided the health benefit to slow the progression of NAFLD and T2DM in obese and pre-diabetic conditions.

## 1 Introduction

Obesity is commonly linked with several metabolic disorders, including non-alcoholic fatty liver disease (NAFLD) and type 2 diabetes mellitus (T2DM). The pathogenesis of obesity also impacts health morbidity and mortality in several aspects; for example, chronic inflammation, cellular oxidative stress, insulin resistance and dyslipidemia (Yang et al., 2022). The primary causes of obesity are physical inactivity with a sedentary lifestyle and high-caloric diet consumption, which lead to an imbalance of energy status. Further progression of excessive fat accumulation in the liver occurs according to increased energy intake and greater body fat storage, especially intra-abdominal fat which increases free fatty acids (FFAs) flux into the liver (Xia et al., 2019), resulting in the development of NAFLD. In addition, insulin resistance affects liver lipid metabolism by enhancing *de novo* lipogenesis and hepatic glucose production (HGP) (Perry et al., 2015; Samuel and Shulman, 2018), which contributes to the further progression of NAFLD as a vicious loop. NAFLD also impacts hepatic insulin action and contributes to the development of T2DM; therefore, the interaction between NAFLD and T2DM plays an important role in developing the consequences of diabetic and hepatic mortalities (Xia et al., 2019).

Recently, bioactive novel metabolites from endophytic fungi have been purposed for therapeutic approaches in many conditions. Endophytic fungi produce secondary metabolites and serve as pharmacological candidates for anticancer, antiviral, and antimicrobial properties (Alurappa et al., 2018; Agrawal et al., 2022). Interestingly, these compounds also provide benefits for the treatment of metabolic disorders (Dompeipen et al., 2011). In our screening of anti-diabetic fungal metabolites employing the glucose uptake induction assay using 3T3-L1 adipocytes, a well-known natural depsidone, nidulin, showed positive results. Therefore, its semisynthetic derivatives, which were previously synthesized from a co-metabolite nornidulin as antibacterial agents (Isaka et al., 2019) were also tested for the assay. One of them, 8-O-(pyridin-4-yl-methyl)nornidulin, given here a trivial name as pyridylnidulin (PN), exhibited the highest glucose uptake-inducing activity (unpublished results). In the present study, we evaluated the metabolic beneficial effects and anti-diabetic properties by using the animal model with obesity and impaired insulin sensitivity.

The mouse models are commonly used to evaluate the effect of bioactive compounds on the therapeutic targets and underlying mechanisms involved in the pathogenesis of NAFLD and T2DM. Diet-induced obesity (DIO) mice are widely used in preclinical research because these mice show similar metabolic characteristics that could be observed in patients (Recena Aydos et al., 2019). Feeding rodents with a high-fat diet (HFD) leads to increased body adiposity and lipid accumulation in the liver, which contributes to developing NAFLD and impaired hepatic insulin sensitivity (Yaligar et al., 2014; Recena Aydos et al., 2019). The alteration in hepatic gene expression of lipid metabolism has been demonstrated after HFD feeding, including the upregulation of sterol regulatory element binding protein-1c (SREBP-1c), fatty acid synthase (FASN), and cluster of differentiation 36 (CD36) gene expression, which involved in liver lipid synthesis and storage (Yang et al., 2012). The hepatic gene expression of gluconeogenesis also increased after HFD, including glucose-6-phosphatase (G6Pc) and phosphoenolpyruvate carboxykinase-1 (Pck-1) (Gan et al., 2022). Taken all together, DIO rodents demonstrate the disorders of lipid and glucose metabolism in the liver following obesogenic HFD feeding.

In this study, we investigated the pharmacological outcomes of PN in mediating liver lipid and glucose metabolism, also the efficacy in glycemic control and systemic adipocytokines that play an important role in regulating insulin sensitivity. In addition, we investigated the underlying mechanisms of PN through which PN promoted health beneficial effects. These findings provided novel drug candidates regarding the slow progression of T2DM and improved insulin sensitivity in pre-diabetic conditions of DIO mice.

## 2 Materials and methods

### 2.1 Synthesis of PN

**FIGURE 1:**
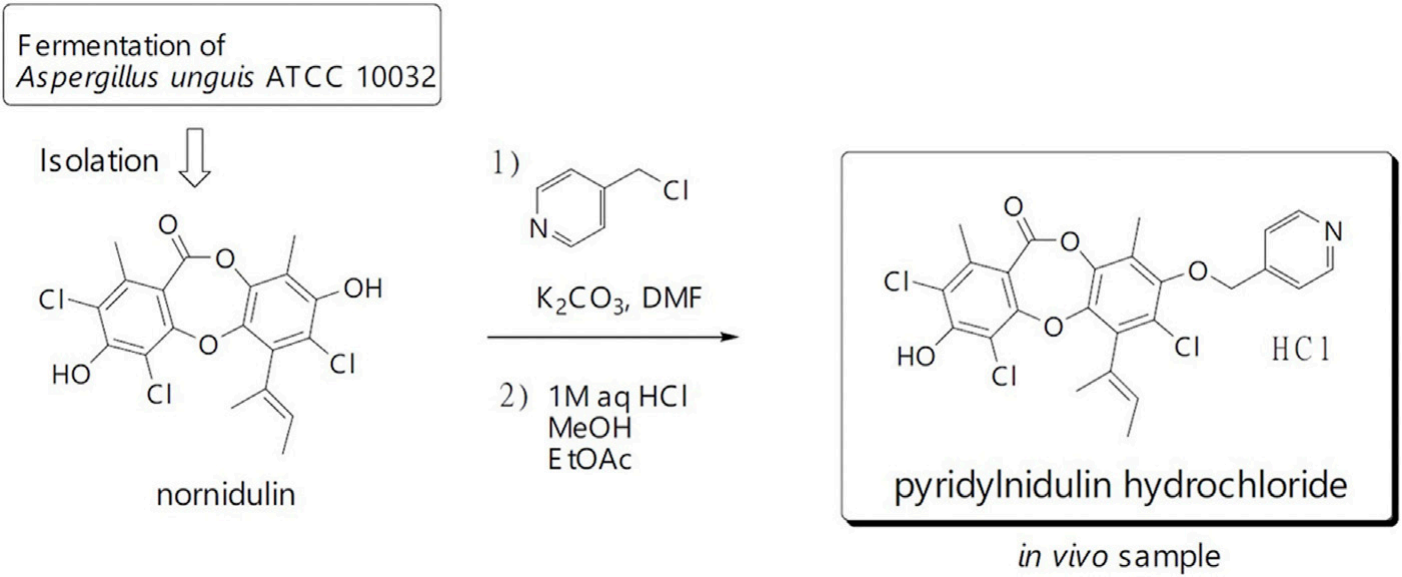
Chemical of pyridylnidulin (PN) hydrochloride and its preparation.

Nornidulin, the substrate of PN, was prepared by repeating large-scale fermentation (50 L) of *Aspergillus unguis* ATCC 10032 and isolation (Isaka et al., 2019). PN was synthesized using the previously reported procedure (Isaka et al., 2019), but on a larger scale (Figure 1). A mixture of nornidulin (4.30 g, 10 mmol), 4-(chloromethyl)pyridine hydrochloride (2.62 mg, 16 mmol), and K_2_CO_3_ (4.15 g, 30 mmol) in *N*,*N*-dimethylformamide (DMF, 30 mL) was stirred at room temperature for 48 h. The mixture was diluted with H_2_O (400 mL) and extracted with ethyl acetate (800 mL). The ethyl acetate layer was washed successively with H_2_O (300 mL × 2), 1M HCl (300 mL), saturated aqueous NaHCO_3_ (300 mL), and saturated aqueous NaCl (200 mL), and then concentrated under reduced pressure to furnish crude reaction products (4.42 g). This synthetic procedure was repeated on a similar scale (nornidulin 4.00 g, 9.3 mmol). The combined crude reaction products (8.58 g) were subjected to silica gel column chromatography (methanol–dichloromethane, step gradient from 0:100 to 4:96) to furnish PN (3.48 g; 35%).

### 2.2 Preparation of PN hydrochloride for animal study

In animal study, PN in hydrochloride form was used due to its greater water solubility. PN (3.48 g, 6.68 mmol) was dissolved in 1M HCl (150 mL), ethyl acetate (150 mL), and methanol (2 mL), the mixture was shaken, and the layer was separated. The bottom (aqueous) layer was extracted with ethyl acetate (150 mL × 2). The combined ethyl acetate layer was concentrated under reduced pressure. The residual solid was dissolved in methanol (40 mL) and concentrated in vacuo furnish PN hydrochloride as a pale-yellow solid (3.26 g, 88%).

### 2.3 Animal model

**FIGURE 2:**
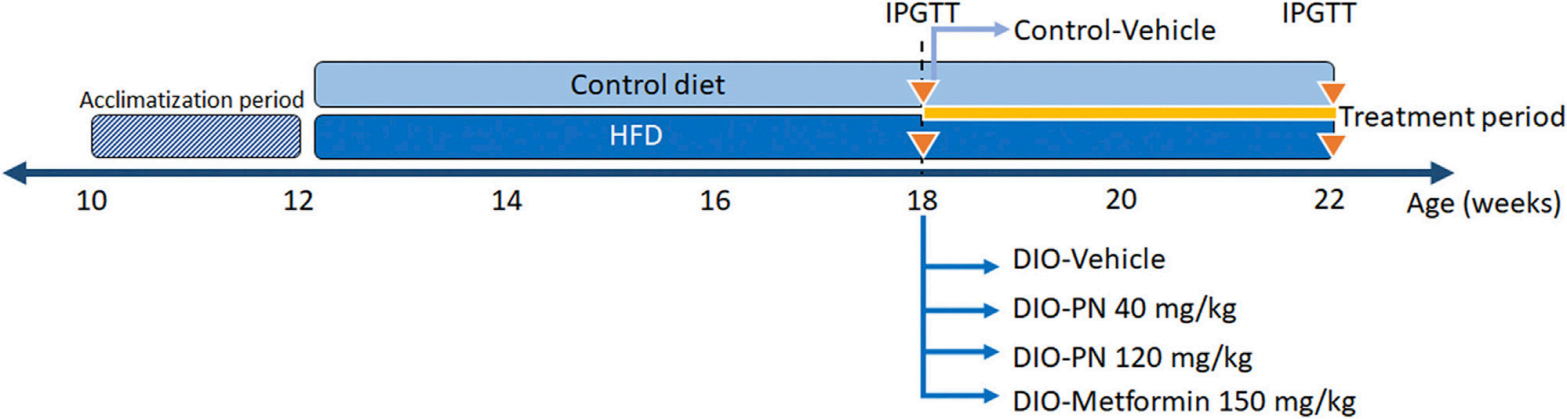
The timeframe of experiment and the treatment groups.

Forty-three male C57Bl/6 mice, aged 10 weeks, from Nomura Siam International Co., Ltd., Bangkok, Thailand were used in this study. The experimental animal use protocol was approved by the Institutional Animal Care and Use Committee (IACUC) of the Faculty of Science, Mahidol University (Protocol No MUSC64–001–550) and consented with the ARRIVE guidelines. All mice were individually housed in a polycarbonate cage (7.5 × 11.5 × 5 inches) under standard conditions in a temperature-controlled room (12/12 h light/dark cycle, 22°C ± 1°C). The mice had access to standard pellet chow containing protein 24 g%, carbohydrate 42 g%, fat 4.5 g%, energy 3.04 kcal/g, and energy from fat 13% (#082; Perfect Companion Group Ltd., Samutprakarn, Thailand) and water *ad libitum*. After acclimatization for 2 weeks, the mice were divided into two groups. The control group (*n* = 8) was fed with the standard pellet chow and the DIO group (*n* = 35) was given an HFD containing protein 20.8%, carbohydrate 41.2%, fat 23.6%, energy 4.60 kcal/g, and energy from fat 45% (TestDiet^®^, Richmond, IN, United States of America) for 6 weeks. Afterward, an intraperitoneal glucose tolerance test (IPGTT) was performed and repeated after treatment (see below). Food intake (22 h ± 0.1 g corrected for spillage) and body weight were recorded daily throughout the experiment. The DIO mice were allocated into 4 groups: 1) DIO-Vehicle (*n* = 8), 2) DIO-PN 40 mg/kg (*n* = 9), 3) DIO-PN 120 mg/kg (*n* = 9), and 4) DIO-Metformin 150 mg/kg (*n* = 9) (#D150959; Sigma-Aldrich, St. Louis, MO, United States), while the control mice were received vehicle, which was the mixture of 5% DMSO, 60% of propylene glycol: polyethylene glycol 400 1:2, 35% of sterile water. All substances were given daily by oral gavage for 4 weeks. The timeframe of the experiment and the treatment groups were represented in Figure 2. After that, the mice were euthanized using xylazine (8 mg/kg; X-LAZINE, L.B.S. Laboratory LTD., Bangkok, Thailand) and the combination drugs of tiletamine HCl and zolazepam HCl (80 mg/kg, Zoletil™100; Virbac, Carros, France). Mice were transcardially perfused with 0.1 M phosphate-buffered saline (PBS) and organ samples were collected, including liver and fat tissue (mesenteric, retroperitoneal and perirenal, epididymal, interscapular, and inguinal fat pads).

### 2.4 Intraperitoneal glucose tolerance test (IPGTT)

After fasting for 12 h, mice were administered with glucose solution (2 g/kg; 50% glucose solution; A.N.B. Laboratories Co., Ltd., Bangkok, Thailand) via intraperitoneal (i.p.) injection. Blood samples were collected by the tail-clipping technique for blood glucose level measurement before glucose administration (fasting blood glucose, FBG) and after 15, 30, 60, 90, and 120 min following glucose injection. Blood glucose levels were measured using a blood glucose meter (Accu-Chek^®^ Performa; Roche Diagnostic GmbH, Mannheim, Germany).

### 2.5 Determination of serum chemistry and lipid profiles

Blood samples were collected from the cardiac puncture. Then, samples were centrifuged at 3,000 *g*, 4°C, for 15 min to obtain the serum. Serum chemistry parameters (for alanine aminotransferase (ALT), aspartate aminotransferase (AST), alkaline phosphatase (ALP)) and lipid profiles (total cholesterol, total triglyceride (TG), high-density lipoprotein cholesterol (HDL-c), low-density lipoprotein cholesterol (LDL-c)) were determined using a chemistry analyzer system (Beckman Coulter AU400, Brea, CA, United States) by enzymatic methods.

### 2.6 Determination of plasma insulin and adipocytokines

Plasma insulin and adipocytokines (leptin, monocyte chemoattractant protein-1 (MCP-1), interleukin-6 (IL-6), tumor necrosis factor-α (TNF-α), plasminogen activator inhibitor-1 (PAI-1)) were measured after treatment. Blood collection was performed by cardiac puncture using K_2_EDTA as an anticoagulant. Then, samples were centrifuged at 3,000 *g*, 4°C, for 15 min to obtain the plasma and stored at −80 °C until the analysis. Plasma insulin and adipocytokines were quantitatively determined using an enzyme-linked immunosorbent assay (ELISA) kit (Milliplex^®^ Mouse Adipokine Magnetic Bead Panel; MilliporeSigma, Burlington, MA, United States) according to the manufacturer’s instructions. Briefly, plasma samples were mixed with magnetic beads coated with antibodies in a 96-black well plate and incubated with overnight shaking at 4 °C. Then, the biotinylated detection antibodies were added and incubated for 30 min. Next, Streptavidin-Phycoerythrin was added and incubated for a further 30 min. After the plate washing steps, Sheath Fluid was added to each well and incubated for 5 min on a plate shaker. Finally, the quantification of fluorescence signals was determined using the Luminex MAGPIX^®^ system (MilliporeSigma, Burlington, MA, United States).

### 2.7 Liver histological study

Liver samples were fixed in 10% neutral buffered formalin (NBF) and embedded in paraffin for histological analysis. Liver sections were stained with hematoxylin and eosin (H&E) and examined the morphology. Evaluation of steatosis and inflammation score according to the NAFLD scoring system for rodents (Liang et al., 2014) was analyzed. The liver photomicrographs were taken using the microscope (Carl Zeiss Microscopy GmbH, Jena, Germany) with Canon EOS Utility Version 2.14.20.0 (Canon Inc., Tokyo, Japan). Semi-quantitative analysis of the average score of steatosis was calculated twice from randomly five different fields (×100 magnification) per mouse.

### 2.8 Liver triglyceride (TG) measurement

Liver samples were collected after perfusion with 0.1 M PBS. The liver samples were then immediately frozen on dry ice and stored at −80 °C until the analysis. Liver TG concentration was measured using a triglyceride quantification kit (#MAK266; Sigma-Aldrich, St. Louis, MO, United States) following the manufacturer’s instructions. Briefly, Liver tissue samples (100 mg) were homogenized in 1 mL of 5% Nonidet™ P40 Substitute (NP40; #74385; Sigma-Aldrich, St. Louis, MO, United States) as described by Huang et al., 2020. Next, liver samples were heated to 80°C–100°C for 2–5 min and cooled to room temperature with this step repeated once, followed by adding lipase to convert TG to glycerol and fatty acids and adding the master reaction mix to each well to complete the reaction. Finally, colorimetric detection was performed by measuring the absorbance at 570 nm.

### 2.9 RNA isolation and quantitative real-time PCR

The extraction of liver RNA was performed using the TRIzol™ reagent (Invitrogen, Carlsbad, CA, United States). Briefly, liver tissue weighing 80 mg was homogenized in 1 mL of TRIzol™ reagent. The separation phase was performed using chloroform (200 µL) and centrifuged at 12,000× *g* at 4°C, for 15 min. Then, the supernatant was collected and transferred to the new microtube. Isopropanol (500 µL) was added to precipitate the RNA and centrifuged at 12,000× *g* at 4°C, for 10 min. The supernatant was discarded, and the RNA pellet was washed with ice-cold 75% ethanol. Samples were then centrifuged at 7,500 *g*, 4°C for 5 min. The RNA pellet was allowed to air dry, while the supernatant was discarded. RNA was redissolved in DNase/RNase-free distilled water (100 µL) (Invitrogen, Carlsbad, CA, United States). The purity of RNA was determined using the Nanodrop 2000C. (Thermo Scientific, Waltham, MA, United States). Synthesis of cDNA utilized the iScript™ cDNA synthesis kit (Bio-Rad, Hercules, CA, United States) according to the manufacturer’s instructions. The amplification of the target gene was detected using SYBR Green (Luna^®^ universal qPCR Master Mix, New England Biolabs, Ipswich, MA, United States), and the specific sequence primers were designed using NCBI/Primer-Blast (Table 1). To evaluate DNA amplification, samples were analyzed using the CFX96™ real-time system (Bio-Rad, Hercules, CA, United States) with Image Lab software (Bio-Rad, Hercules, CA, United States). The relative expression levels of gene transcription were analyzed using the 2^−ΔΔCt^ method and normalized with GAPDH.

**TABLE 1:** The sequence primers for quantitative RT-PCR

Sterol regulatory element binding protein-1c (SREBP-1c)	Forward (5’→3′)	CAA​GGC​CAT​CGA​CTA​CAT​CCG
	Reverse (5’→3′)	CAC​CAC​TTC​GGG​TTT​CAT​GC
Fatty acid synthase (FASN)	Forward (5’→3′)	GGA​GTT​CTC​AGG​CCG​GGA​TA
	Reverse (5’→3′)	GGG​TAC​ATC​CCA​GAG​GAA​GTC​A
Acetyl CoA carboxylase α (Acaca)	Forward (5’→3′)	ATG​GGC​GGA​ATG​GTC​TCT​TTC
	Reverse (5’→3′)	TGG​GGA​CCT​TGT​CTT​CAT​CAT
Cluster of differentiation 36 (CD36)	Forward (5’→3′)	GCT​CGT​TTC​AAC​TCT​CAC​ACA​C
	Reverse (5’→3′)	ACG​TGG​CCC​GGT​TCT​ACT​AAT​TC
Glucose-6-phosphatase (G6Pc)	Forward (5’→3′)	TGG​TAG​CCC​TGT​CTT​TCT​TT
	Reverse (5’→3′)	TCA​GTT​TCC​AGC​ATT​CAC​AC
Phosphoenolpyruvate carboxykinase-1 (Pck-1)	Forward (5’→3′)	GAT​GTC​GGA​AGA​GGA​CTT​TGA​G
	Reverse (5’→3′)	CAT​AGG​GCG​AGT​CTG​TCA​GTT​C
Glucokinase (GCK)	Forward (5’→3′)	ACA​GTC​TCC​TTC​ATA​TAC​CTC​CAC
	Reverse (5’→3′)	CTC​TAT​CCT​CTG​GCA​TCT​CCT
GAPDH	Forward (5’→3′)	CAA​GCT​CAT​TTC​CTG​GTA​TGA​CA
	Reverse (5’→3′)	GCC​TCT​CTT​GCT​CAG​TGT​CC

### 2.10 Western blot analysis

The frozen liver samples were prepared in an ice-cold RIPA buffer (Cell Signaling Technology, Danvers, MA, United States) containing protease inhibitors (Cell Signaling Technology, Danvers, MA, United States) and phosphatase inhibitors (PhosSTOP™, Merck Millipore, Darmstadt, Germany). Samples were homogenized using MiniBeadBeater-16 (BioSpec Products, Bartlesville, OK, United States) with stainless steel bead 3.2 mm. Then, samples were centrifuged at 12,000× *g* at 4°C, for 10 min to remove cell debris. The supernatant was used to measure the protein concentration using the BCA protein assay kit (Merck Millipore, Darmstadt, Germany). Proteins were separated using 8%–10% sodium dodecyl sulfate-polyacrylamide gel electrophoresis (SDS-PAGE) and transferred to a polyvinylidene fluoride (PVDF) membrane. The membrane was blocked from non-specific binding using 2% BSA for 1 h. Following the blocking step, membranes were incubated overnight at 4 °C with primary antibodies: *ß*-actin (#4970), fatty acid synthase (FAS #3180), Acetyl Co-A Carboxylase (ACC #3662), p-ACC (Ser79) (#3661), AMP-activated protein kinase-α (AMPK-α #2532), p-AMPK-α (Thr172) (#2535) (Cell Signaling Technology^®^, Danvers, MA, United States). After washing with tris-buffered saline with Tween-20 (TBST), membranes were incubated with anti-rabbit IgG HRP-linked antibody (#7074, Cell Signaling Technology^®^, Danvers, MA, United States) After washing with TBST, the specific protein bands were developed by incubation for 4 min with ECL substrate (Immobilon^®^ Forte Western HRP Substrate; #WBLUF0100; EMD Millipore, Burlington, MA, United States). Then, membranes were exposed to a GelDoc XR imaging system (Bio-Rad, Hercules, CA, United States) and the band intensity was analyzed using Image Lab 6.1 software (Bio-Rad, Hercules, CA, United States). The results were normalized with the densitometry of *ß*-actin.

### 2.11 Statistical analysis

The results were presented as mean ± standard error of the mean (SEM). Significant different values were considered as *p* ≤ 0.05. Analysis of the parameters between control and DIO mice was assessed by an independent *t*-test. Analysis of statistically significant difference among the treatment groups was performed using one-way analysis of variance (ANOVA). A Bonferroni test was used for *post hoc* analysis. Data analysis was evaluated with GraphPad Prism 7.00 (GraphPad Software, San Diego, CA, United States).

## 3 Results

### 3.1 Characteristics of diet-induced obesity (DIO) mice

**FIGURE 3:**
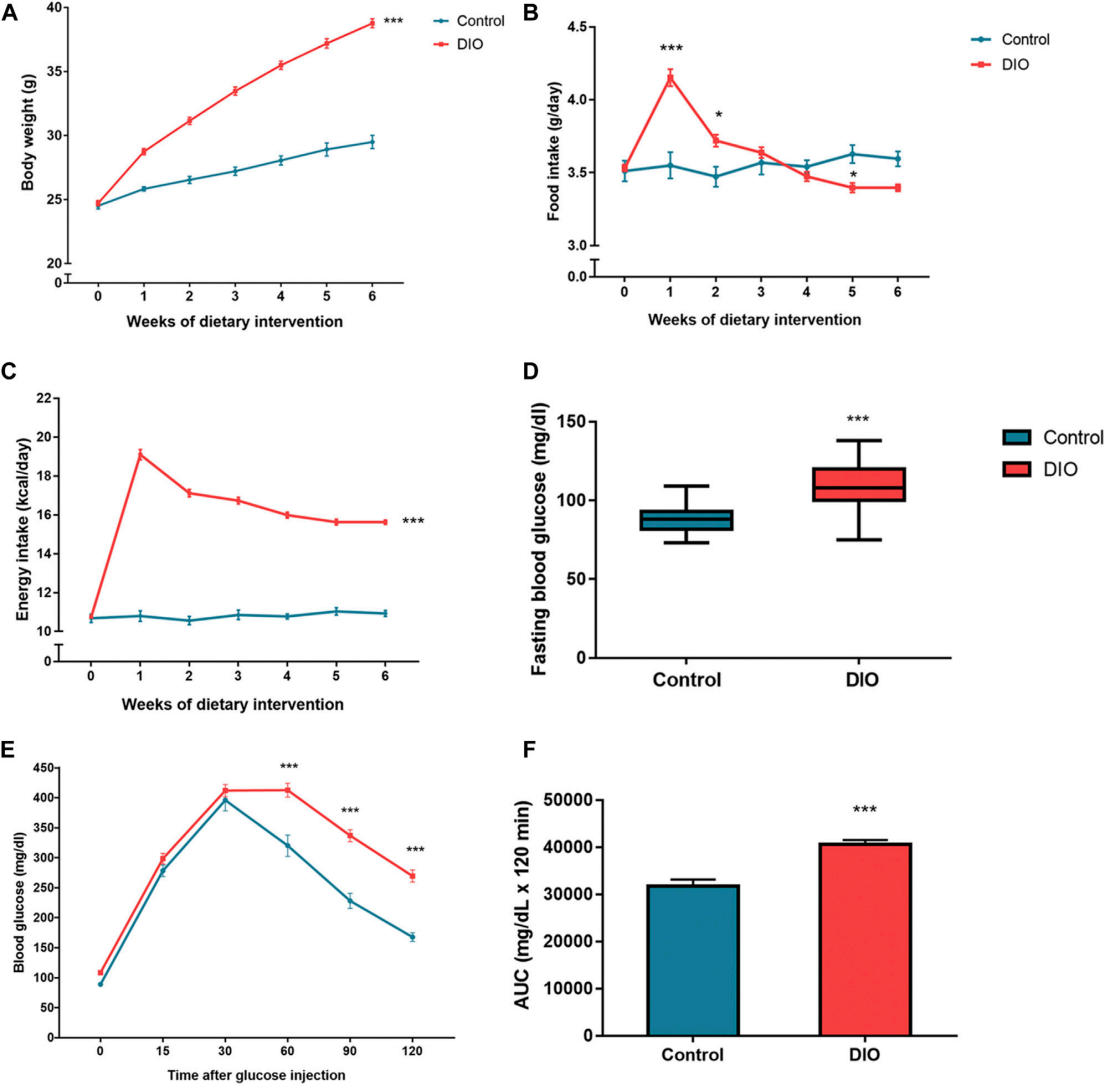
**(A)** The body weight of the control and DIO mice during dietary intervention for 6 weeks; *** represents statistical significance compared to control mice (*p* < 0.001). **(B)** Food intake of the control and DIO mice during dietary intervention for 6 weeks; * represents statistical significance compared to control mice (*p* < 0.05), *** represents statistical significance compared to control mice (*p* < 0.001). **(C)** Energy intake of the control and DIO mice during dietary intervention for 6 weeks; *** represents statistical significance compared to control mice (*p* < 0.001). **(D)** Fasting blood glucose of the control and DIO mice after HFD feeding; *** represents statistical significance compared to control mice (*p* < 0.001). **(E)** Intraperitoneal glucose tolerance test (IPGTT) in control and DIO mice; *** represents statistical significance compared to control mice (*p* < 0.001). **(F)** Area under the curve (AUC) of IPGTT; *** represents statistical significance compared to control mice (*p* < 0.001).

The DIO mice were developed after a 6-week HFD induction. The body weight (BW) of DIO mice showed a significant difference as compared to control mice (*p* < 0.001) (Figure 3A). DIO mice had a significant difference in the daily FI which showed a greater amount, especially during the first and second weeks of dietary intervention (*p* < 0.001 and *p* < 0.05, respectively), when compared to the control mice (Figure 3B); however, the significant trend of the lowered amount of FI was found in the later period. The energy intake (EI) was shown statistical significance in DIO mice throughout the dietary intervention period (*p* < 0.001) (Figure 3C). In addition, DIO mice also showed significantly higher fasting blood glucose (FBG) (*p* < 0.001) (Figure 3D) and impaired glucose tolerance (*p* < 0.001) (Figures 3E,F) compared to control mice. These results indicated that DIO mice developed the pre-diabetic condition with obesity.

### 3.2 Effects of PN on FI, glucose tolerance, serum biochemistry parameters and lipid profiles

**FIGURE 4:**
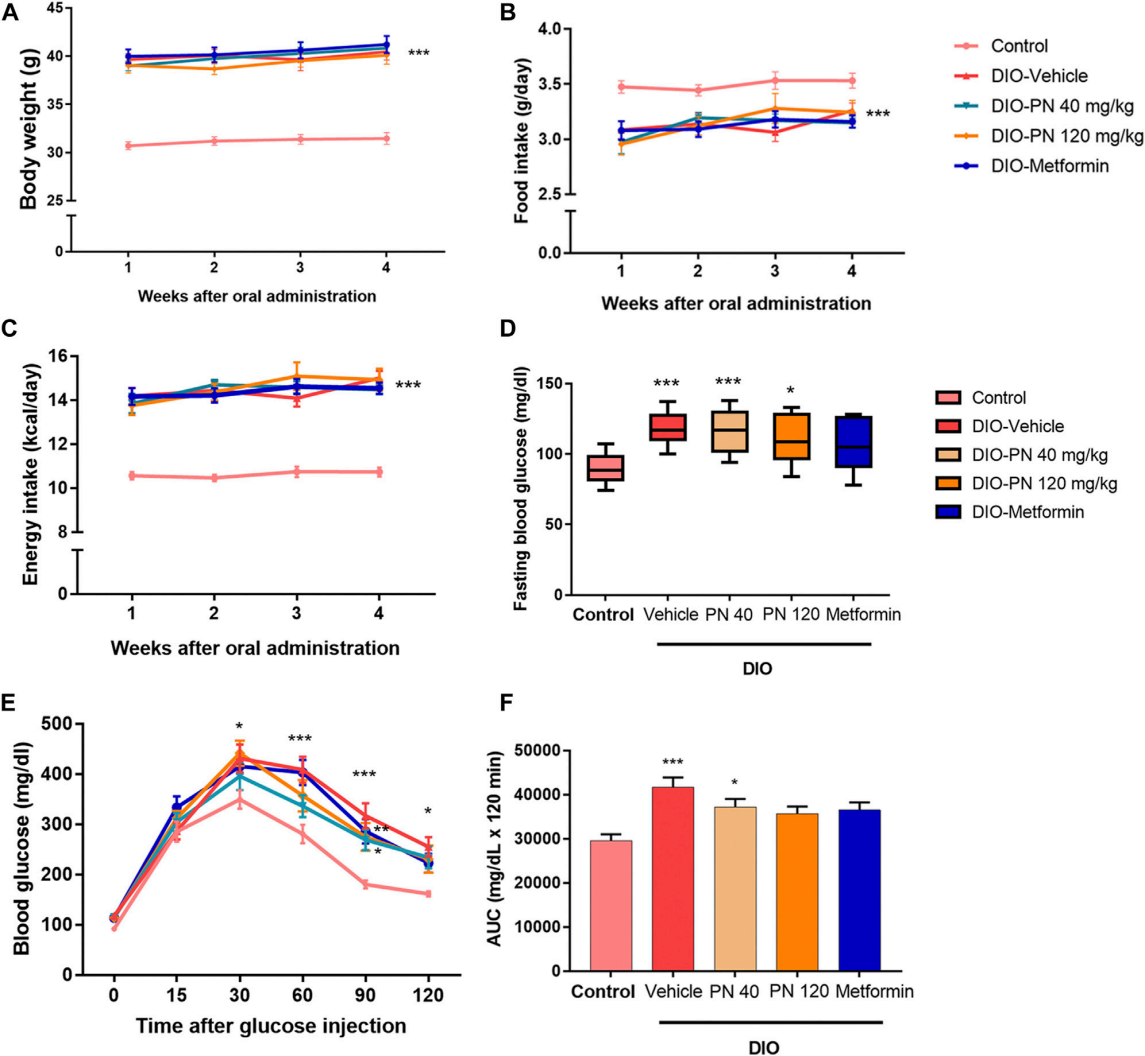
**(A)** The body weight of the control and DIO mice during oral administration for 4 weeks; *** represents statistical significance compared to control mice (*p* < 0.001). **(B)** Food intake of the control and DIO mice during oral administration for 4 weeks; *** represents statistical significance compared to control mice (*p* < 0.001). **(C)** Energy intake of the control and DIO mice during oral administration for 4 weeks; *** represents statistical significance compared to control mice (*p* < 0.001). **(D)** Fasting blood glucose of the control and DIO mice after oral administration; * represents statistical significance compared to control mice (*p* < 0.05), *** represents statistical significance compared to control mice (*p* < 0.001). **(E)** Intraperitoneal glucose tolerance test (IPGTT) in control and DIO mice; * represents statistical significance compared to control mice (*p* < 0.05), ** represents statistical significance compared to control mice (*p* < 0.01), *** represents statistical significance compared to control mice (*p* < 0.001). **(F)** Area under the curve (AUC) of IPGTT; * represents statistical significance compared to control mice (*p* < 0.05), *** represents statistical significance compared to control mice (*p* < 0.001).

**FIGURE 5:**
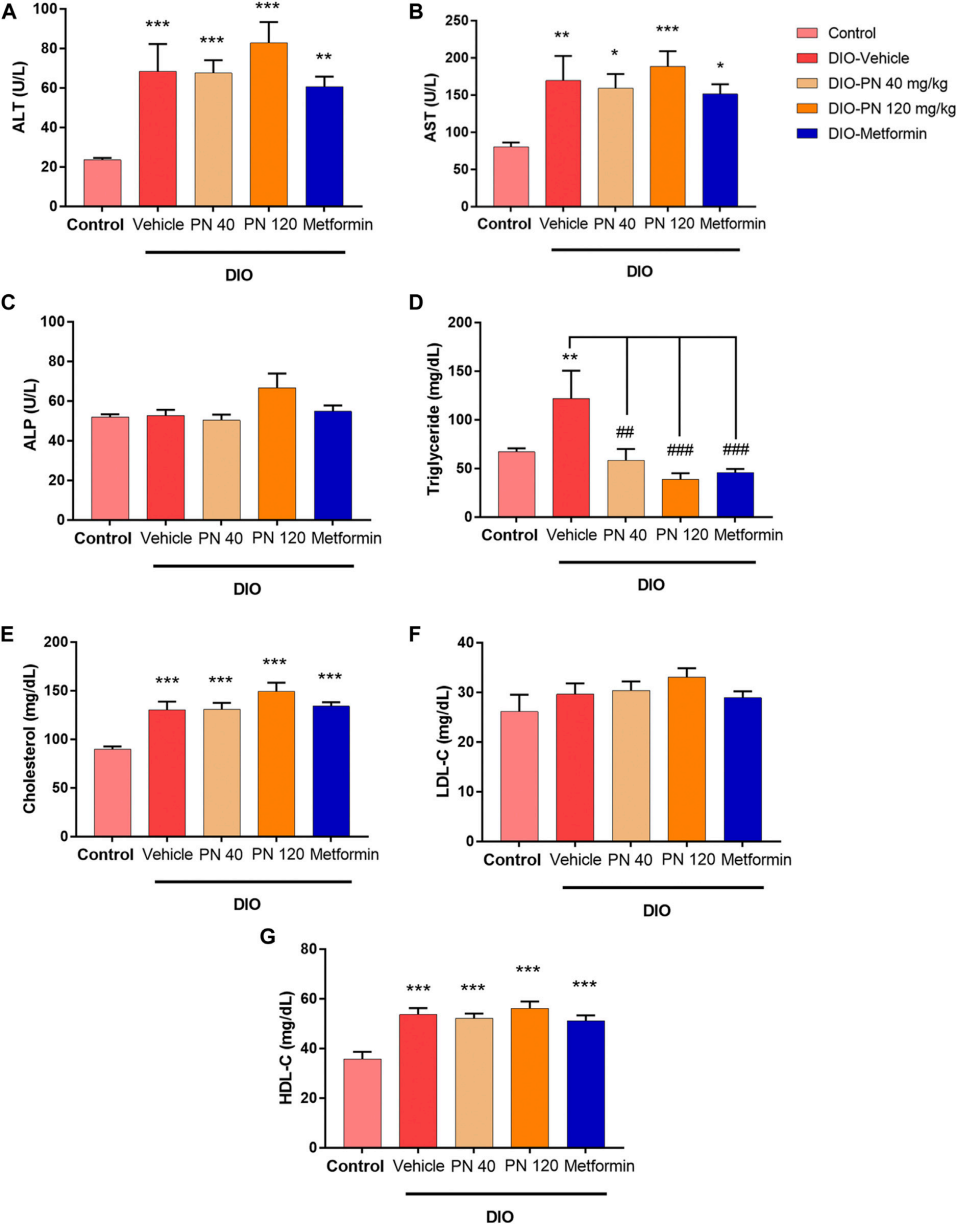
**(A)** Serum alanine aminotransferase enzyme (ALT); ** represents statistical significance compared to control mice (*p* < 0.01), *** represents statistical significance compared to control mice (*p* < 0.001). **(B)** Serum aspartate aminotransferase enzyme (AST); * represents statistical significance compared to control mice (*p* < 0.05), ** represents statistical significance compared to control mice (*p* < 0.01), *** represents statistical significance compared to control mice (*p* < 0.001). **(C)** Serum alkaline phosphatase enzyme (ALP). **(D)** Serum triglyceride (TG) levels; ** represents statistical significance compared to control mice (*p* < 0.01), ^##^ represents statistical significance compared to DIO-vehicle mice (*p* < 0.01), ^###^ represents statistical significance compared to DIO-vehicle mice (*p* < 0.001). **(E)** Serum cholesterol levels; *** represents statistical significance compared to control mice (*p* < 0.001). **(F)** Serum low-density lipoprotein cholesterol (LDL-c) levels. **(G)** Serum high-density lipoprotein cholesterol (HDL-c); *** represents statistical significance compared to control mice (*p* < 0.001).

During the oral administration of PN (40 and 120 mg/kg), there were no changes in BW, FI, and EI (Figures 4A–C). After 4 weeks of oral administration, improved glucose tolerance was shown in the high dose of PN (120 mg/kg) and metformin-treated mice (Figures 4D–F). Body adiposity was determined after the treatment period, and the results of each site of adipose tissue depot were shown in the Supplementary Material (Supplementary Figure S1). Serum biochemistry parameters were shown in Figure 5. Liver enzymes (ALT, AST) were higher in DIO mice (*p* < 0.001 and *p* < 0.01, respectively), while PN administration, at any dose, did not alter these parameters (Figures 5A,B). Although the low dose of PN-treated mice (40 mg/kg) tended to lower serum AST, it was not significantly different when compared to vehicle-treated DIO mice (Figure 5B). The levels of ALP were shown in Figure 5C, there was no statistically significant difference in DIO and control mice (*p* > 0.05). Serum TG level was higher in DIO mice (*p* < 0.01) which was improved by PN and metformin treatment (Figure 5D). In addition, a higher level of serum cholesterol was found in DIO mice (*p* < 0.001) compared to the control group, neither PN nor metformin improved the serum cholesterol level (Figure 5E). The levels of LDL-c were not statistically different in control and DIO mice with any treatment (Figure 5F). However, the levels of HDL-c were greater in DIO mice compared to the control group (*p* < 0.05), while PN administration, at any dose, was not decreased LDL-c level (Figure 5G).

### 3.3 Effects of PN on plasma insulin and adipocytokine levels

**FIGURE 6:**
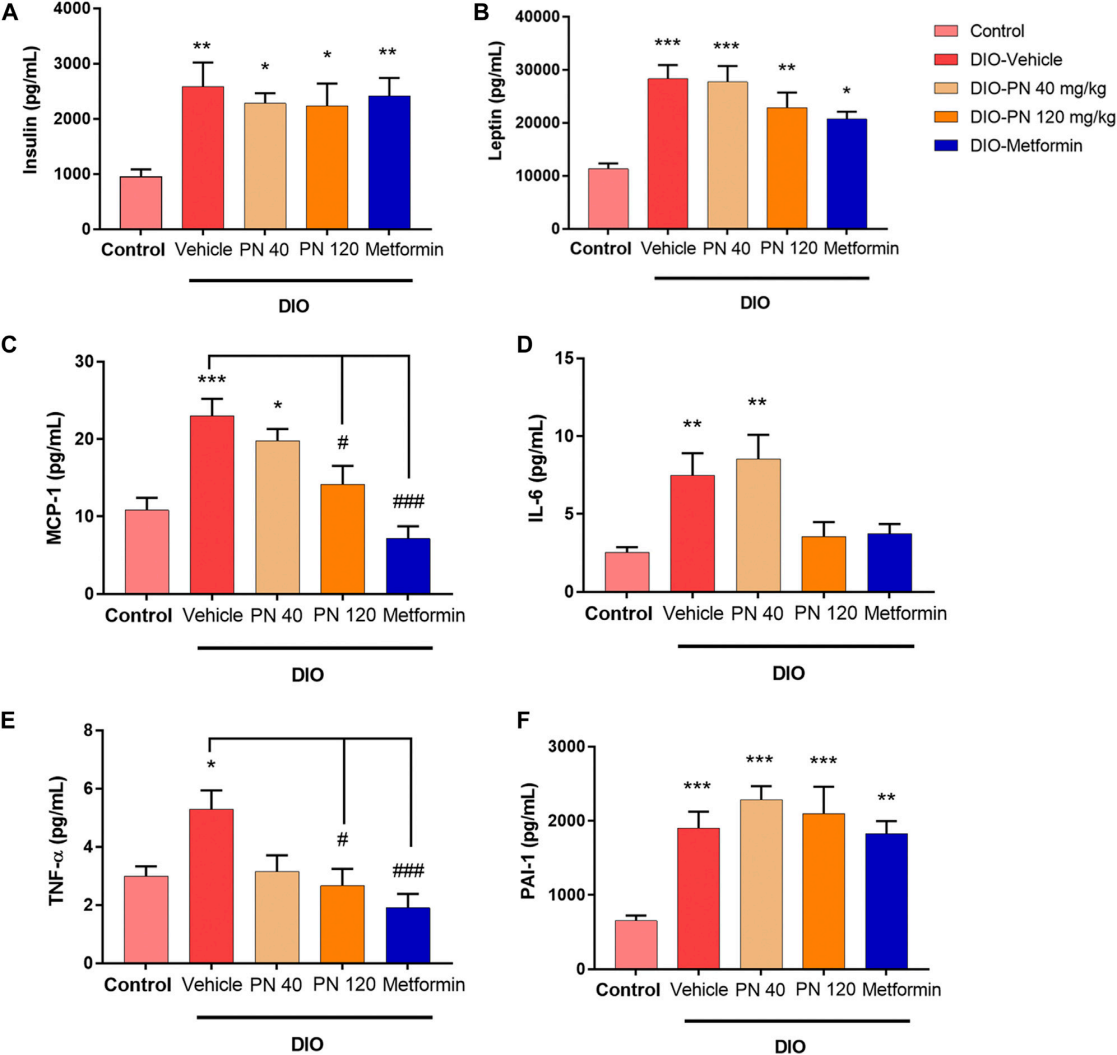
**(A)** Plasma insulin levels; * represents statistical significance compared to control mice (*p* < 0.05), ** represents statistical significance compared to control mice (*p* < 0.01). **(B)** Plasma leptin levels; * represents statistical significance compared to control mice (*p* < 0.05), ** represents statistical significance compared to control mice (*p* < 0.01), *** represents statistical significance compared to control mice (*p* < 0.001). **(C)** Plasma monocyte chemoattractant protein-1 (MCP-1) levels; * represents statistical significance compared to control mice (*p* < 0.05), *** represents statistical significance compared to control mice (*p* < 0.001), ^#^ represents statistical significance compared to DIO-vehicle mice (*p* < 0.05), ^###^ represents statistical significance compared to DIO-vehicle mice (*p* < 0.001). **(D)** Plasma interleukin-6 (IL-6) levels; ** represents statistical significance compared to control mice (*p* < 0.01). **(E)** Plasma tumor necrosis factor-α (TNF-α) levels; * represents statistical significance compared to control mice (*p* < 0.05), ^#^ represents statistical significance compared to DIO-vehicle mice (*p* < 0.05), ^###^ represents statistical significance compared to DIO-vehicle mice (*p* < 0.001). **(F)** Plasminogen activator inhibitor-1 (PAI-1); ** represents statistical significance compared to control mice (*p* < 0.01), *** represents statistical significance compared to control mice (*p* < 0.001).

The plasma concentrations of insulin and adipocytokines (leptin, MCP-1, IL-6, TNF-α, and PAI-1) were shown in Figure 6. Plasma insulin levels were higher in DIO mice compared to control mice (*p* < 0.01) which was in accordance with metformin-treated mice. For PN-treated mice, at any dose, plasma insulin levels were statistically significant when compared to control mice (*p* < 0.05) (Figure 6A). In addition, plasma leptin levels were higher in DIO mice and the low-dose PN-treated (40 mg/kg) mice compared to control mice (*p* < 0.001). Increased plasma leptin levels were also shown in the high-dose PN-treated (120 mg/kg) and metformin-treated mice (*p* < 0.01 and *p* < 0.05, respectively) (Figure 6B). The levels of plasma MCP-1 were increased in DIO mice compared to control mice (*p* < 0.001). Decreased plasma MCP-1 levels were shown in the high-dose PN-treated (120 mg/kg) and metformin-treated mice (*p* < 0.05 and *p* < 0.001, respectively) compared to the DIO-vehicle group (Figure 6C). The levels of pro-inflammatory cytokines (IL-6 and TNF-α) were higher in DIO mice (*p* < 0.01 and *p* < 0.05, respectively), while plasma IL-6 level was higher in low-dose PN-treated (40 mg/kg) mice (*p* < 0.01) compared to control mice (Figure 6D). Decreased plasma TNF-α levels were shown in the high-dose PN-treated (120 mg/kg) and metformin-treated mice (*p* < 0.05 and *p* < 0.001, respectively) compared to the DIO-vehicle group (Figure 6E). In addition, the levels of plasma PAI-1 were higher in the vehicle, PN, and metformin-treated DIO mice (*p* < 0.001 except metformin group, and *p* < 0.01 for metformin-treated mice) compared to control mice (Figure 6F). These results indicated that the high dose of PN (120 mg/kg) and metformin potentiated to reduce of the adipocytokines related to the pathogenesis of T2DM.

### 3.4 Effects of PN on liver steatosis score, liver TG levels, and liver inflammation score

**FIGURE 7:**
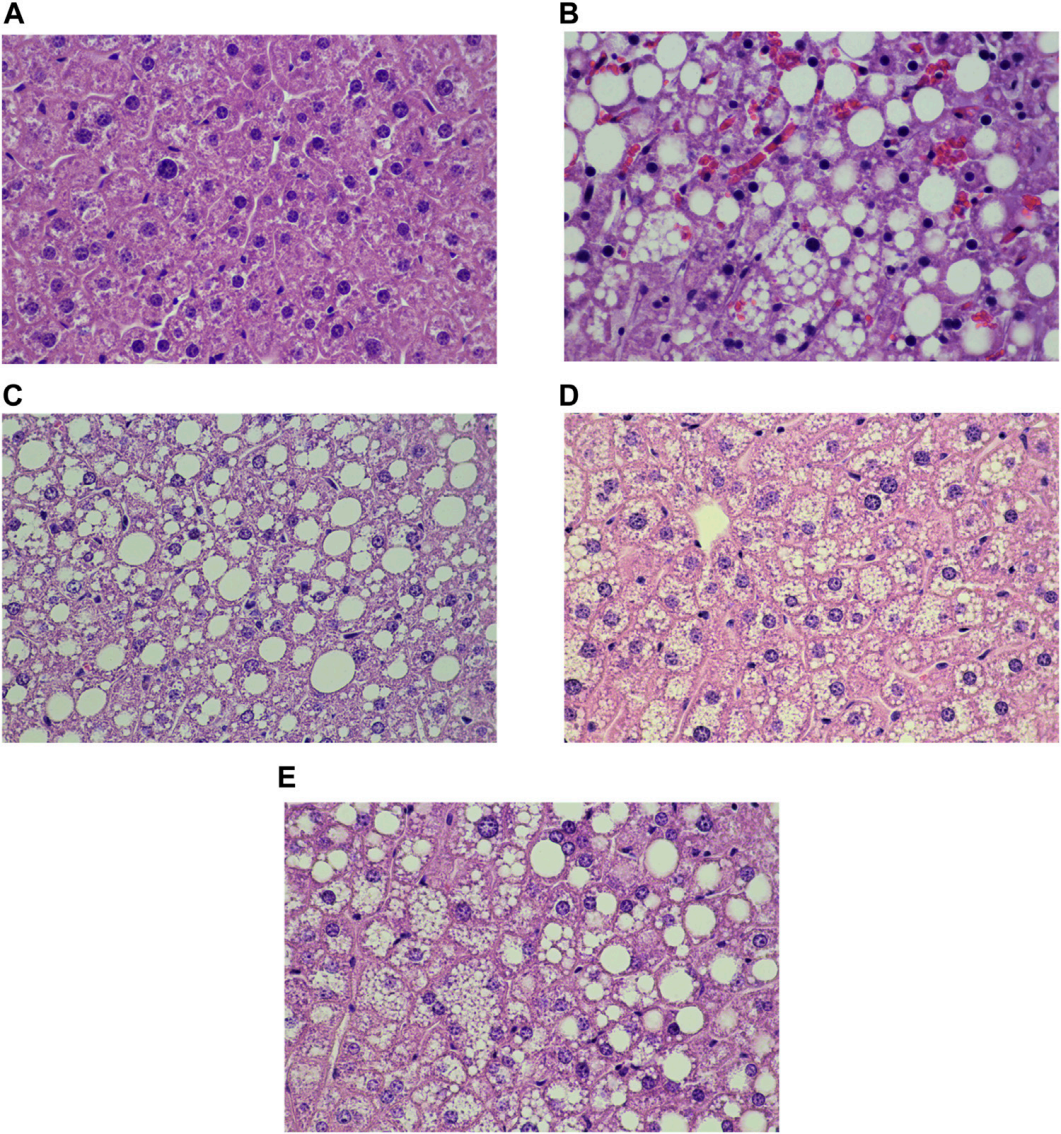
Liver histology of the control and DIO mice (Hematoxylin and eosin staining; x 40x); **(A)** control mice, **(B)** DIO-vehicle, **(C)** DIO-PN 40 mg/kg, **(D)** DIO-PN 120 mg/kg, **(E)** DIO-metformin.

**FIGURE 8:**
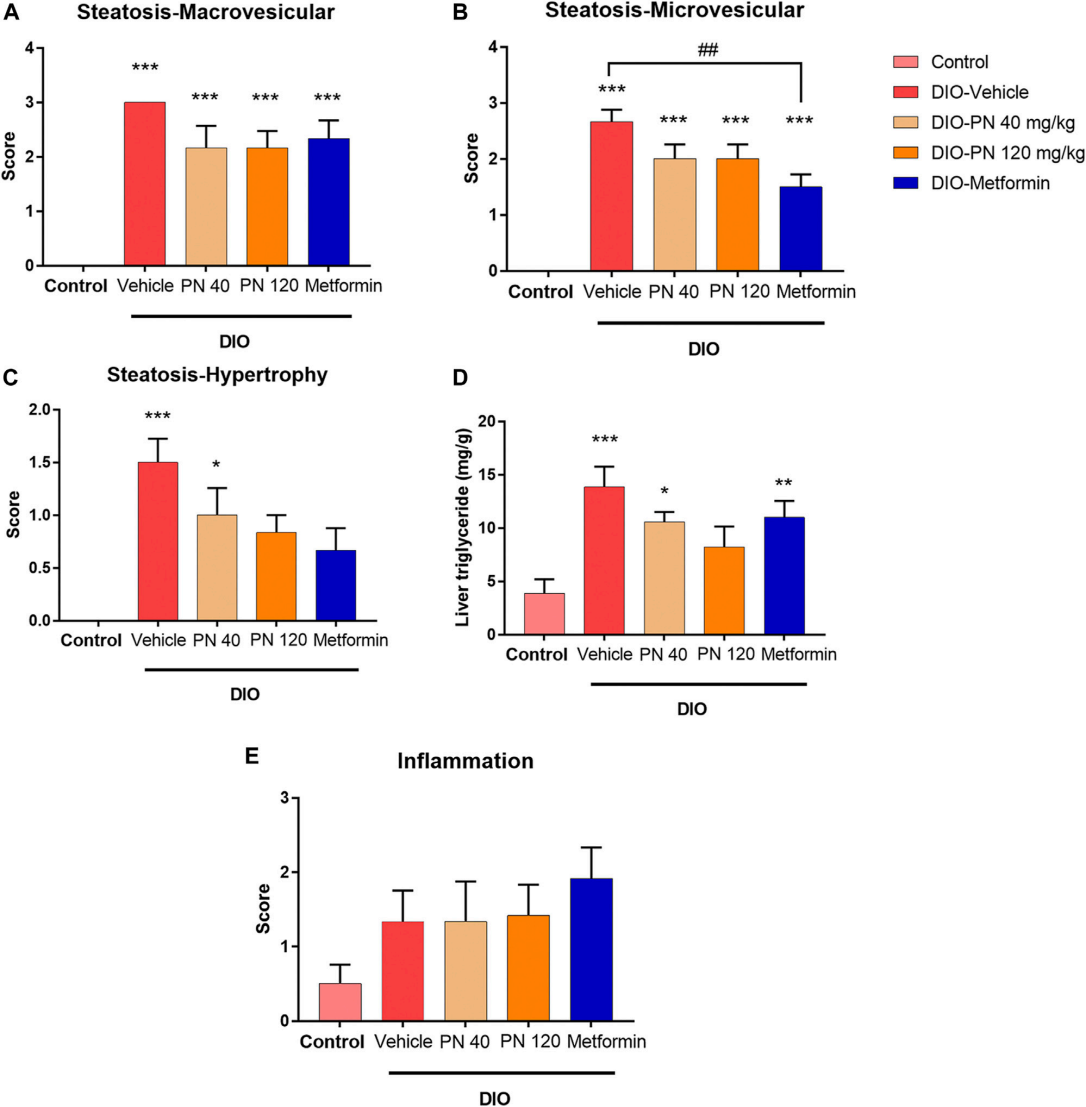
**(A)** Liver steatosis score—macrovesicular steatosis (large lipid droplet); *** represents statistical significance compared to control mice (*p* < 0.001). **(B)** Liver steatosis score—microvesicular steatosis (small lipid droplet); *** represents statistical significance compared to control mice (*p* < 0.001), ^##^ represents statistical significance between DIO-vehicle and DIO-metformin (*p* < 0.01). **(C)** Liver steatosis score—cellular hypertrophy (enlargement >1.5x of the normal hepatocyte diameter); * represents statistical significance compared to control mice (*p* < 0.05), *** represents statistical significance compared to control mice (*p* < 0.001). **(D)** Liver triglyceride levels; * represents statistical significance compared to control mice (*p* < 0.05), ** represents statistical significance compared to control mice (*p* < 0.01), *** represents statistical significance compared to control mice (*p* < 0.001). **(E)** Liver inflammation score.

Liver histology and scoring of steatosis and inflammation were performed as shown in Figures 7, 8, respectively. Liver steatosis score determined macrovesicular steatosis which revealed the presence of large lipid droplets or vacuoles displaced the nucleus to the side, while microvesicular steatosis was the small size of lipid droplets present in hepatocytes. The hepatocellular hypertrophy determined the enlarged hepatocyte was more than 1.5x of normal. Liver inflammation determined the cluster of more than 5 inflammatory cells in 10 different fields. The results showed that DIO mice had a greater score of macro- and microvesicular steatosis (*p* < 0.001), while the PN administered, at any dose, had not decreased the steatosis score (Figures 8A,B). In addition, metformin decreased microvesicular steatosis (*p* < 0.01) compared to the DIO-vehicle group (Figure 8B). In addition, vehicle- and low-dose PN (40 mg/kg) treated DIO mice had significantly increased hepatocellular hypertrophy (*p* < 0.001 and *p* < 0.05, respectively) compared to control mice (Figure 8C). The levels of liver TG were consistent with the result of hepatocellular hypertrophy as shown in Figure 8D. Vehicle- and low-dose PN (40 mg/kg) treated DIO mice had significantly increased liver TG levels (*p* < 0.001 and *p* < 0.05, respectively). However, metformin-treated mice also significantly increased liver TG (*p* < 0.01) compared to control mice (Figure 8D). For the liver inflammation score, there were no statistically significant differences among all groups (*p* > 0.05, Figure 8E).

### 3.5 Effects of PN on liver mRNA expression involved in lipid and glucose metabolism

**FIGURE 9:**
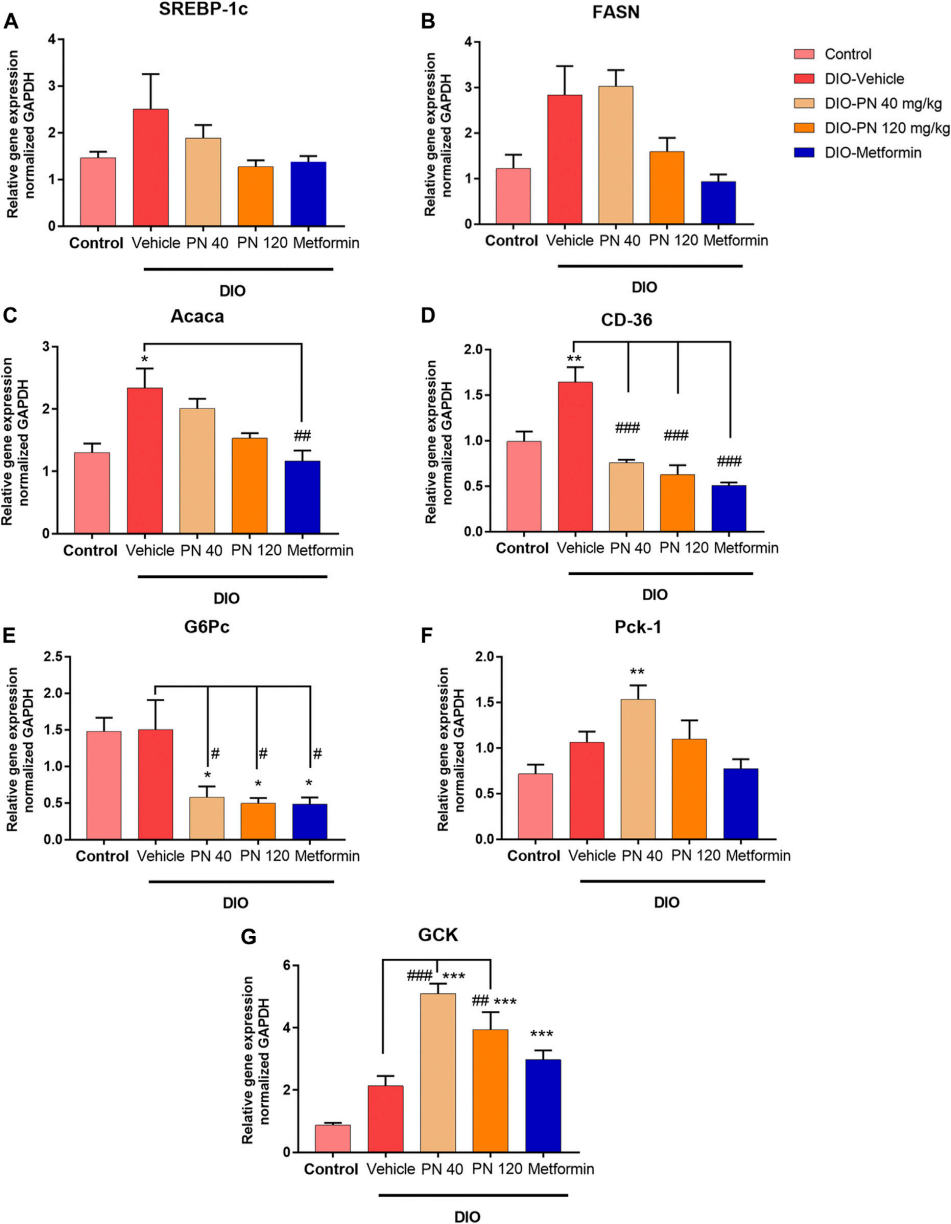
Liver mRNA expression **(A)** Sterol regulatory element binding protein-1c (SREBP-1c). **(B)** Fatty acid synthase (FASN). **(C)** Acetyl CoA carboxylase α (Acaca); * represents statistical significance compared to control mice (*p* < 0.05), ^##^ represents statistical significance compared to DIO-vehicle mice (*p* < 0.01). **(D)** Cluster of differentiation 36 (CD36); ** represents statistical significance compared to control mice (*p* < 0.01), ^###^ represents statistical significance compared to DIO-vehicle mice (*p* < 0.001). **(E)** Glucose-6-phosphatase (G6Pc); * represents statistical significance compared to control mice (*p* < 0.05), ^#^ represents statistical significance compared to DIO-vehicle mice (*p* < 0.05). **(F)** Phosphoenolpyruvate carboxykinase-1 (Pck-1); ** represents statistical significance compared to control mice (*p* < 0.01). **(G)** Glucokinase (GCK); *** represents statistical significance compared to control mice (*p* < 0.001), ^##^ represents statistical significance compared to DIO-vehicle mice (*p* < 0.01), ^###^ represents statistical significance compared to DIO-vehicle mice (*p* < 0.001).

The liver mRNA expression associated with lipid metabolism, including SREBP-1c, FASN, Acaca, and CD36, was shown in Figures 9A–D. The upregulation of SREBP-1c and FASN expression was found in the vehicle- and low-dose PN (40 mg/kg) treated DIO mice; however, it did not reach a significant difference when compared to control mice (*p* > 0.05) (Figures 9A,B). The high dose of PN (120 mg/kg) and metformin tended to downregulate the mRNA expression but did not reach statistically significant results (*p* > 0.05). In addition, the expression of Acaca was upregulated in the DIO-vehicle group (*p* < 0.05) when compared to the control group. Administration of PN, with any doses, did not affect the expression level, while metformin-treated mice showed the downregulation of Acaca mRNA expression (*p* < 0.01) when compared to vehicle-treated DIO mice (Figure 9C). The expression of CD36 was in accordance with Acaca, which was increased in vehicle-treated DIO mice (*p* < 0.01) when compared to control mice. Interestingly, administration of PN, with any doses, and metformin provided a highly significant effect on the downregulation of CD36 mRNA expression (*p* < 0.001) when compared to vehicle-treated DIO mice (Figure 9D). In addition, the liver mRNA expression involved in glucose metabolism, including G6Pc, Pck-1, and GCK, was shown in Figures 9E–G. The expression of G6Pc was downregulated in PN and metformin-treated mice (*p* < 0.05) when compared to control and vehicle-treated DIO mice (Figure 9E). By contrast, the expression of Pck-1 was upregulated in the low dose of PN (40 mg/kg) treated mice (*p* < 0.01) when compared to control mice (Figure 9F). For the expression of GCK, upregulation of GCK mRNA expression was shown in PN, with any doses, and metformin-treated mice (*p* < 0.001) when compared to control mice. Regarding the effect of PN administered, GCK mRNA expression was upregulated in the low dose of PN (40 mg/kg) and the high dose of PN (120 mg/kg) treated mice (*p* < 0.001 and *p* < 0.01, respectively) when compared to vehicle-treated DIO mice (Figure 9G).

### 3.6 Effects of PN on the liver protein expression involved in lipid metabolism and potentiated to improve glucose intolerance

**FIGURE 10:**
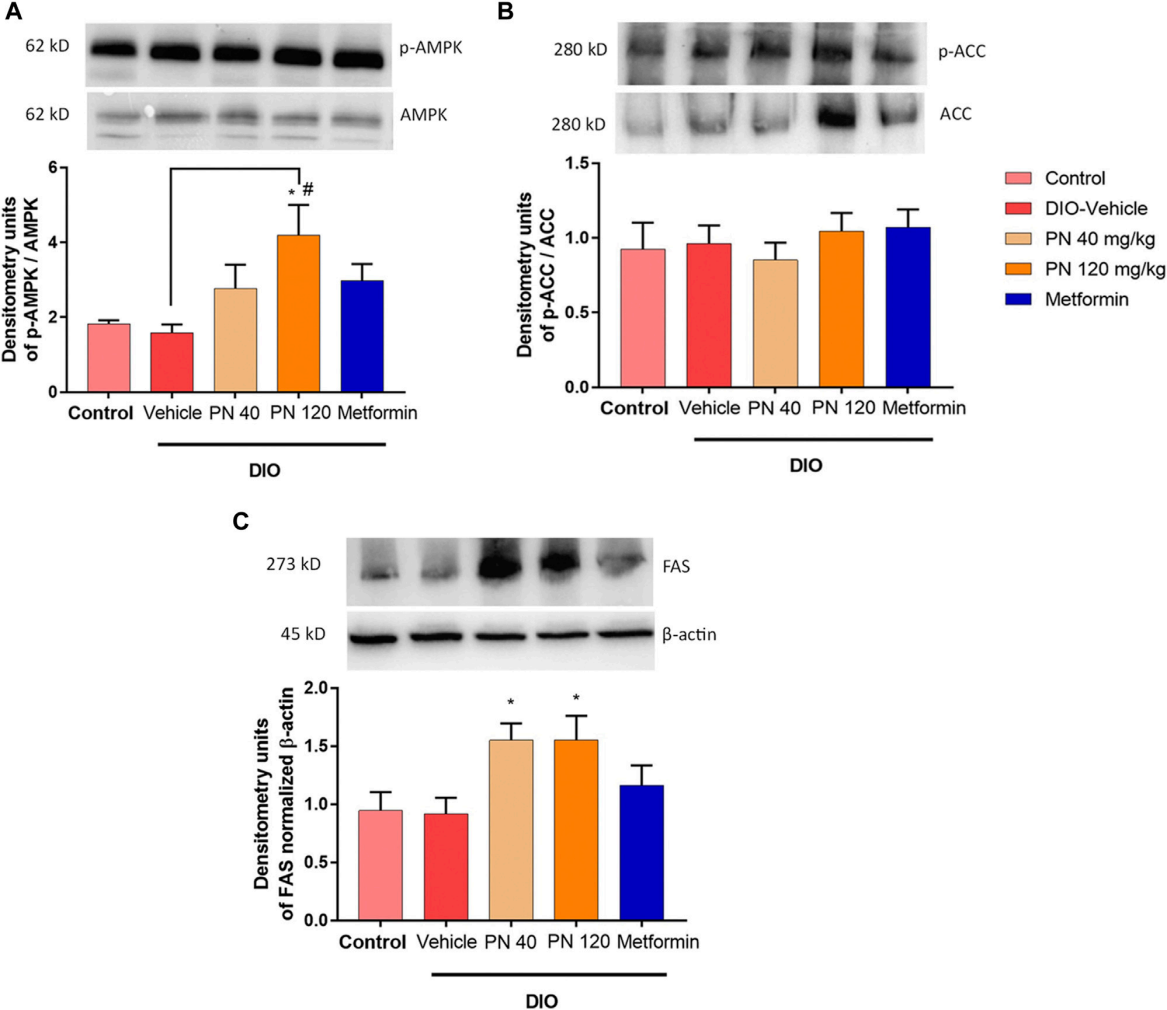
**(A)** Liver p-AMPK/AMPK protein levels; * represents statistical significance compared to control mice (*p* < 0.05), ^#^ represents statistical significance compared to DIO-vehicle mice (*p* < 0.05). **(B)** Liver p-ACC/ACC protein levels. **(C)** Liver FAS protein levels; * represents statistical significance compared to control mice (*p* < 0.05).

Oral administration of PN for 4 weeks increased the expression of p-AMPK/AMPK, especially in the high dose of PN (120 mg/kg) (*p* < 0.05), when compared to control and vehicle-treated DIO mice (Figure 10A). However, the low dose of PN (40 mg/kg) and metformin-treated mice tended to show increased p-AMPK/AMPK expression, but it did not reach statistically significant levels (*p* > 0.05). The expression of p-ACC/ACC was not shown statistically significant between groups (*p* > 0.05, Figure 10B). In addition, the expression of FAS was increased by PN administration, with any doses (*p* < 0.05, Figure 10C), when compared to the control group.

## 4 Discussion

This present study reported the potentiated role of the anti-diabetic effect of fungal metabolite, which played an important role in drug discovery and development. PN was the candidate for the bioactive compound metabolite which provided health benefits in several aspects. First, PN regulated the normal glycemic control, and acted as the euglycemic compound, with improved systemic insulin sensitivity. Second, PN improved liver lipid metabolism by transcriptional regulation, resulting in reduced liver TG levels, in turn reducing lipid export as the decreased serum TG levels. Lastly, PN decreased the levels of adipocytokines, especially MCP-1 and TNF-α, which played an important role in the low-grade inflammatory state related to impaired insulin sensitivity and further contributed to the development of T2DM.

Insulin resistance was the key mechanism of T2DM. This state was characterized by impaired insulin-stimulated glucose uptake from the insulin target tissue (skeletal muscle and adipose tissue) and insufficient suppression of hepatic gluconeogenesis (Wilcox, 2005). Hepatic insulin resistance was related to increased hepatic glucose production and *de novo* lipogenesis, resulting in hyperglycemia, NAFLD, and dyslipidemia (Meshkani and Adeli, 2009). In addition, NAFLD shared common pathophysiological features with T2DM of insulin resistance and excessive lipid accumulation (Mu et al., 2018; Guo et al., 2021). In our study, we demonstrated the effects of oral PN administration regarding the glucose metabolism and lipid metabolism, especially in the liver, using DIO mice. In accordance with the previous studies, DIO mice were induced with HFD feeding, resulting in impaired glucose tolerance and higher level of FBG (Della Vedova et al., 2016; Appiakannan et al., 2020; Guo et al., 2021). Interestingly, the results revealed that metformin and PN improved glucose intolerance and decreased fasting blood glucose. These results suggested that PN was considered in anti-diabetic drug development.

The pathophysiology of T2DM patients was related to impaired glucose homeostasis, resulting in hyperglycemia. The major contributing factors consisted of increased hepatic glucose production and decreased hepatic glucose utilization (Rines et al., 2016; Kim et al., 2020). GCK, the key enzyme that regulates glucose utilization and metabolism, played the role in the phosphorylation of glucose into glucose-6-phosphate which was no longer transported to blood circulation. The hepatic gene expression of GCK was insulin dependent (Matschinsky and Wilson, 2019). In the present study, GCK gene expression was upregulated in PN and metformin-treated mice. In addition, hepatic glucose production was regulated by G6Pc and Pck-1. Pck-1 was the rate-limiting gluconeogenic enzyme that converts oxaloacetate to phosphoenolpyruvate (Ye et al., 2023), while G6Pc was required for the final step of gluconeogenesis which released glucose and phosphate group (Hatting et al., 2018). Insulin also regulated the gene transcription of G6Pc and Pck-1 (Hatting et al., 2018). Our results demonstrated that PN and metformin reduced the gene expression of G6Pc but did not alter the gene expression of Pck-1. On the contrary, the low dose of PN-treated mice increased the gene expression of Pck-1, which might be due to the low dose of PN-treated mice having impaired glucose tolerance and developing an insulin-resistant state. A previous study reported that Pck-1 activated the lipogenesis pathway (Xu et al., 2020), thus it might be the complex regulator interplay between gluconeogenesis and lipogenesis. Taken together, PN, especially the high dose, contributed to the glucose-lowering effect by regulating glucose utilization and gluconeogenic gene expression levels.

Liver lipid metabolism played an important role in developing NAFLD and T2DM. The accumulation of lipids in the liver was acquired through fatty acid uptake and *de novo* lipogenesis (Ipsen et al., 2018). Insulin resistance increased *de novo* lipogenesis (Leclercq et al., 2007), which converted acetyl Co-A to fatty acids and eventually stored as TG. The key transcription factor controlling *de novo* lipogenesis was SREBP-1c, which was upregulated by insulin (Ipsen et al., 2018). The downstream pathway of SREBP-1c was associated with increased Acaca and FASN gene transcription. In our present study, PN-treated mice, especially the high dose, decreased the liver TG levels which was in accordance with downregulated gene transcription of SREBP-1c, Acaca, and FASN; however, the expression level did not reach statistical significance. Fatty acid uptake contributed to the development of NAFLD. Fatty acid translocase (CD36) gene expression was related to insulin resistance, obesity, and liver steatosis (Zeng et al., 2022). Our results showed that PN and metformin-treated mice significantly promoted the downregulation of CD36 gene expression. Assessment of liver steatosis score revealed that PN and metformin-treated mice significantly reduced hepatocellular hypertrophy, which was one of the steatosis criteria. Metformin also reduced microvesicular steatosis, which provided the benefit regarding improved NAFLD. In addition, PN and metformin-treated mice significantly decreased the levels of serum TG which were beneficial to improving the metabolic profiles, although serum cholesterol levels were unaffected in this study. However, our study did not induce steatohepatitis in DIO mice and the liver inflammation score was not altered with any treatment.

Insulin resistance was associated with chronic low-grade inflammation. The accumulation of excessive fat produced and secreted adipocytokines, which interfered with insulin signaling pathways, resulting in the progression of insulin resistance and T2DM (Kwon and Pessin, 2013). Leptin was released from adipocytes in proportion to body adiposity. Leptin can induce monocytes and macrophages to produce pro-inflammatory cytokines, including IL-6 and TNF-α (Gainsford et al., 1996). IL-6 was positively correlated with obesity; however, the role of IL-6 was controversial, as IL-6 disrupted insulin signaling pathways, resulting in insulin resistance (Senn et al., 2003). Whereas IL-6 deficient mice developed insulin resistance and liver inflammation (Matthews et al., 2010). MCP-1 was responsible for the increase in macrophages which contributed to inducing an inflammatory state and insulin resistance (Rehman and Akash, 2016). TNF-α stimulated various transcriptional pathways, including nuclear factor kappa-B (NF-κB) and Jun NH2-terminal kinase (JNK), which impaired insulin signaling pathways (Rehman and Akash, 2016). PAI-1 acted as a negative regulator of fibrinolysis by inhibiting tissue plasminogen activator (Yarmolinsky et al., 2016). It should be noted that the levels of PAI-1 were associated with impaired glucose tolerance and insulin resistance (Festa et al., 1999). In our present study, plasma leptin levels tended to decrease in the high dose of PN and metformin-treated mice but did not reach statistical significance. The levels of IL-6 were higher in the low dose of PN which may contribute to the insulin-resistant state and impaired glucose tolerance, while IL-6 levels were lower in the high dose of PN and metformin-treated mice. The levels of MCP-1 were consistent with TNF-α in the high dose of PN and metformin-treated mice, which were significantly decreased when compared to vehicle-treated DIO mice. In addition, the levels of PAI-1 were unaffected by PN and metformin treatment. Our results revealed that the decreased levels of TNF-α, IL-6, and MCP-1, especially in the high-dose of PN and metformin-treated mice could be suggested that reduced the inflammatory state which further improved insulin sensitivity.

The underlying molecular metabolism that PN exerted beneficial effects on hyperglycemia, insulin sensitivity, and NAFLD progression was demonstrated in this study. The activation of AMPK acted as the negative regulator of *de novo* fatty acid synthesis and cholesterol biosynthesis (Agius et al., 2020). Liver-specific activation of AMPK potentiated the inhibition of liver TG accumulation (Woods et al., 2017). The previous study reported that metformin-mediated AMPK activation promoted the suppression of SREBP-1c and eventually inhibited fatty acid synthesis (Kaneto et al., 2021). In addition, metformin-mediated AMPK activation reduced hepatic gluconeogenesis by the suppression of Pck-1 and G6Pc (Kaneto et al., 2021). In our present study, we demonstrated that PN activated the AMPK signaling pathway to modulate glucose homeostasis and liver lipid metabolism. Unfortunately, the protein expression levels of ACC and FAS were not consistent with these results. The levels of ACC protein expression were unaffected, but the levels of FAS protein expression were higher in PN-treated mice. The post-translational modification of proteins following protein biosynthesis should be considered and further study required to investigate the ACC and FAS enzyme activity.

## 5 Conclusion

**FIGURE 11:**
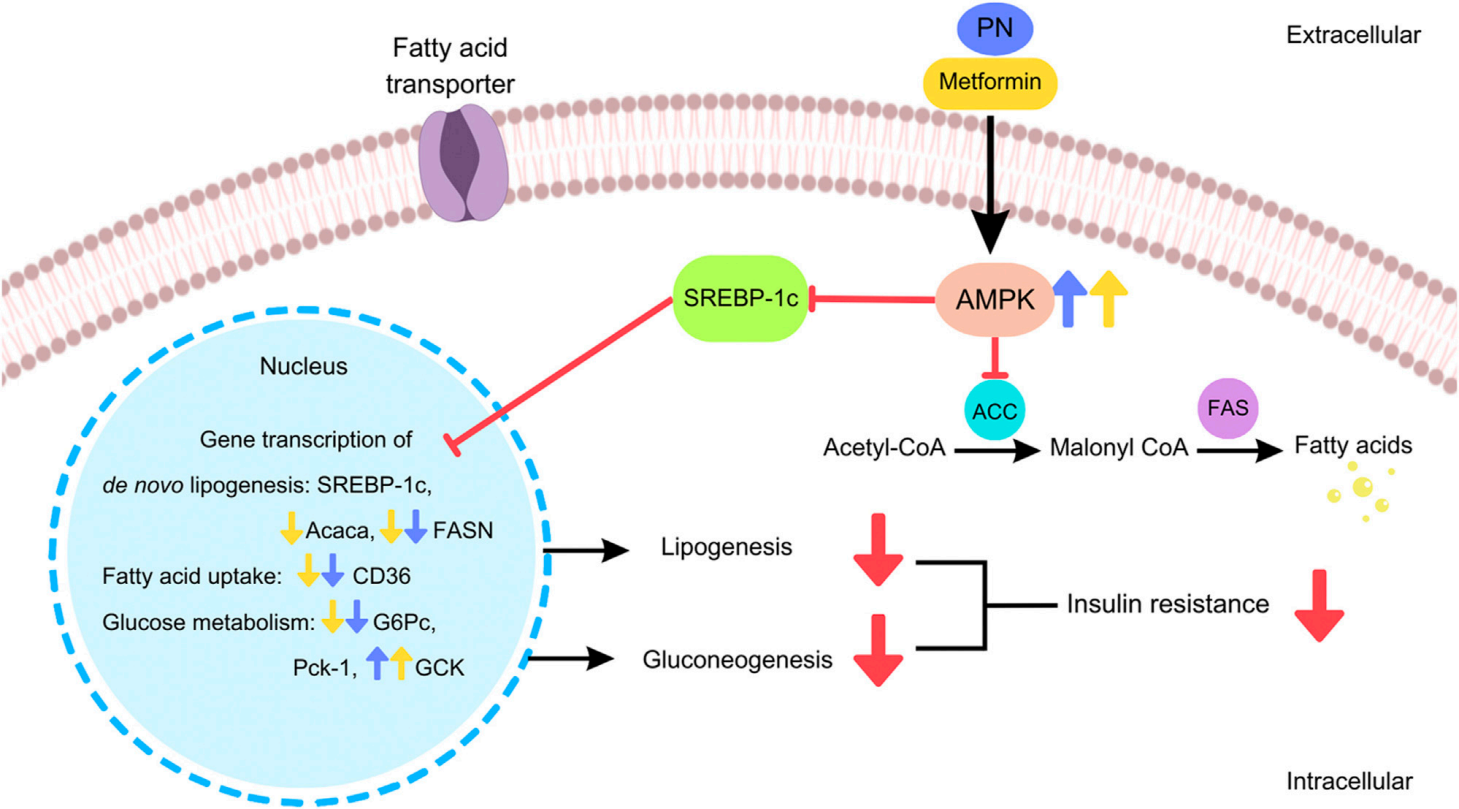
The cellular signaling pathways of PN and metformin regulated the hepatic gene transcription and exerted beneficial effects in DIO mice.

The results of the present study demonstrated that the oral administration of PN regulated the gene transcription of liver lipid metabolism in DIO mice by the downregulation of the gene involved in *de novo* lipogenesis (FASN) and fatty acid uptake (CD36), resulting in the reduction of liver TG. In addition, PN administration provided a beneficial way to improve insulin sensitivity by the downregulation of the gene involved in gluconeogenesis (G6Pc) and the upregulation of the gene that encoded the key enzyme in glucose utilization (GCK). The stimulation via the AMPK signaling pathway was the underlying mechanism of PN to improve insulin sensitivity and lipid profiles (Figure 11). These findings supported the concept that PN acted as an insulin sensitizer compound.

## Data Availability

The original contributions presented in the study are included in the article/Supplementary Material, further inquiries can be directed to the corresponding author.
