# Supplementary material for: Pyridylnidulin exerts anti-diabetic properties and improves non-alcoholic fatty liver disease in diet-induced obesity mice
Source: Front Mol Biosci. 2023 Jun 22;10:1208215. doi: 10.3389/fmolb.2023.1208215 (PMC10324605; doi:10.3389/fmolb.2023.1208215)
Supplement: Supplementary file 1 [file DataSheet1.DOCX]

Supplementary Material

Pyridylnidulin exerts anti-diabetic properties and improves non-alcoholic fatty liver disease in diet-induced obesity mice

Sutharinee Likitnukul *, Surapun Tepaarmorndech, Theerayuth Kaewamatawong, Arunrat Yangchum, Chanathip Duangtha, Pimrapat Jongjang, Supachoke Mangmool, Darawan Pinthong, Masahiko Isaka

*** Correspondence:** Sutharinee Likitnukul: sutharinee.lik@mahidol.ac.th

# Supplementary Figures and Tables

## Supplementary Figures

**
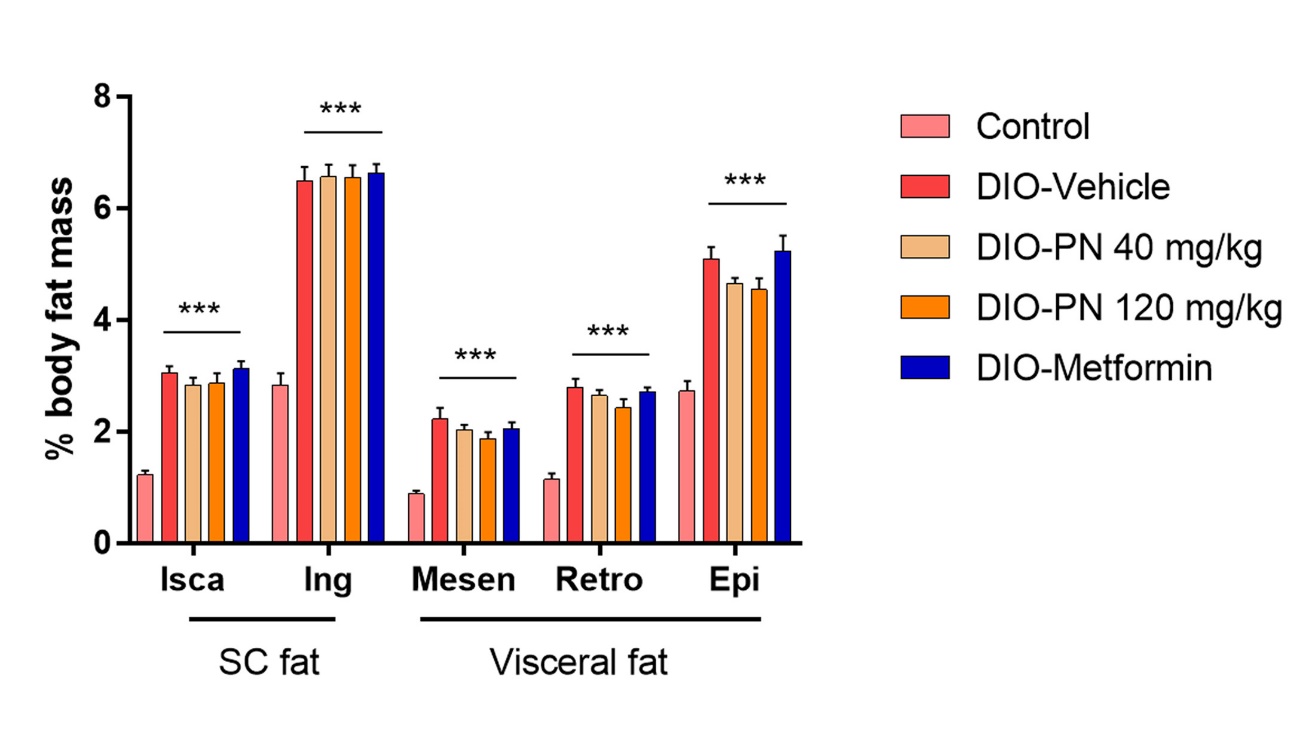
**

**Supplementary Figure 1.** The percentage of body fat mass in the mice; *** represents statistical significance when compared to control group (*p* < 0.001). Isca: interscapular adipose tissue, Ing: inguinal adipose tissue, Mesen: mesenteric adipose tissue, Retro: retroperitoneal and perirenal adipose tissue, Epi: epididymal adipose tissue.
